# Cardiovascular magnetic resonance imaging in the UK Biobank: a major international health research resource

**DOI:** 10.1093/ehjci/jeaa297

**Published:** 2020-11-09

**Authors:** Zahra Raisi-Estabragh, Nicholas C Harvey, Stefan Neubauer, Steffen E Petersen

**Affiliations:** 1 William Harvey Research Institute, Centre for Advanced Cardiovascular Imaging, NIHR Barts Biomedical Research Centre, Queen Mary University of London, Charterhouse Square, London EC1M 6BQ, UK; 2 Barts Heart Centre, Department of Cardiac Imaging, St. Bartholomew’s Hospital, Barts Health NHS Trust, London EC1A 7BE, UK; 3 MRC Lifecourse Epidemiology Unit, University of Southampton, Southampton, SO16 6YD, UK; 4 NIHR Southampton Biomedical Research Centre, University of Southampton and University Hospital Southampton NHS Foundation Trust, Southampton, SO16 6YD, UK; 5 Division of Cardiovascular Medicine, Radcliffe Department of Medicine, National Institute for Health Research Oxford Biomedical Research Centre, University of Oxford, Oxford, OX3 9DU, UK

**Keywords:** UK Biobank, cardiovascular magnetic resonance, epidemiology, population health, big data

## Abstract

The UK Biobank (UKB) is a health research resource of major international importance, incorporating comprehensive characterization of >500 000 men and women recruited between 2006 and 2010 from across the UK. There is prospective tracking of health outcomes for all participants through linkages with national cohorts (death registers, cancer registers, electronic hospital records, and primary care records). The dataset has been enhanced with the UKB imaging study, which aims to scan a subset of 100 000 participants. The imaging protocol includes magnetic resonance imaging of the brain, heart, and abdomen, carotid ultrasound, and whole-body dual X-ray absorptiometry. Since its launch in 2015, over 48 000 participants have completed the imaging study with scheduled completion in 2023. Repeat imaging of 10 000 participants has been approved and commenced in 2019. The cardiovascular magnetic resonance (CMR) scan provides detailed assessment of cardiac structure and function comprising bright blood anatomic assessment (sagittal, coronal, and axial), left and right ventricular cine images (long and short axes), myocardial tagging, native T1 mapping, aortic flow, and imaging of the thoracic aorta. The UKB is an open access resource available to health researchers across all scientific disciplines from both academia and industry with no preferential access or exclusivity. In this paper, we consider how we may best utilize the UKB CMR data to advance cardiovascular research and review notable achievements to date.

## Introduction to the UK Biobank

The UK Biobank (UKB) comprises a cohort of >500 000 men and women aged 40–69 years at recruitment (2006–10). Baseline assessment included a comprehensive series of questionnaires, face-to-face interviews, physical measures, and blood sampling. The full protocol is publicly available^1^ and summary data may be viewed on the UKB website: www.ukbiobank.ac.uk. Blood biomarker (haematology and biochemistry) and whole-genome sequencing are available for all participants (released 2019). The UKB imaging study was launched in 2015, with the aim of scanning 20% of the original cohort, that is, 100 000 participants.^2^ The imaging protocol includes magnetic resonance imaging of the brain, heart, and abdomen, carotid ultrasound, and whole-body dual X-ray absorptiometry. To date (September 2020), >48 000 participants have completed the imaging study with scheduled completion by the end of 2023. Repeat imaging of 10 000 participants commenced in 2019 and is also due for completion in 2023. Selected components of the baseline assessment were repeated for a subset of 20 000 participants between 2012 and 2013 (calibration visit) and at both imaging visits, permitting adjustment for random measurement error and estimation of longitudinal variations.

Health outcomes for all UKB participants are prospectively tracked through linkages with electronic hospital records, cancer registers, death registers, and primary care records. The UKB has also produced algorithmically defined outcomes for incidence of key illnesses, such as myocardial infarction, through cross-checking over multiple data sources.^3^ The scale of the UKB and the indefinite follow-up of participants means that there should be sufficient numbers of a wide range of incident illnesses for adequately powered nested case–control studies (*Table 1*)^1^ and indeed for prospective cohort analyses for more common outcomes. The documentation of incident outcomes some years after assessment of exposures reduces (although does not remove completely) the chance of reverse causation explaining observed associations. In addition, whilst there is, as is usual with such cohorts, evidence of healthy selection; there is, for the majority of variables, a substantial range of risk factor levels and disease rates within the UKB population, with sufficient variation to allow adequately powered analyses, which may be generalizable across a range of demographics.^4^^,^^5^

**Table 1:** Estimated number of years from baseline to accrue cases of selected conditions in UK Biobank^a^

	Time to achieve (years)				
	1000 cases	2500 cases	5000 cases	10 000 cases	20 000 cases
MI and coronary death	2	4	5	8	13
Stroke	5	8	12	18	28
Diabetes mellitus	2	3	4	6	10
COPD	4	6	8	13	23
Colorectal cancer	5	9	14	22	42
Hip fracture	7	11	15	21	31
Alzheimer’s disease	7	10	13	18	23
Parkinson’s disease	6	10	15	23	37

Adapted from Ref.^1^

COPD, chronic obstructive pulmonary disease; MI, myocardial infarction.

a Estimated years from start of recruitment in 2006 with allowance for healthy cohort effect, overseas migration and comprehensive withdrawal of 1 in 500 participants.

The UKB is an open access resource available to health researchers across all scientific disciplines from both academia and industry with no preferential access or exclusivity. New researchers can find details on formal access procedures (including the modest access charges based on a cost recovery model) on the UKB website: www.ukbiobank.ac.uk.

**Figure 1:**
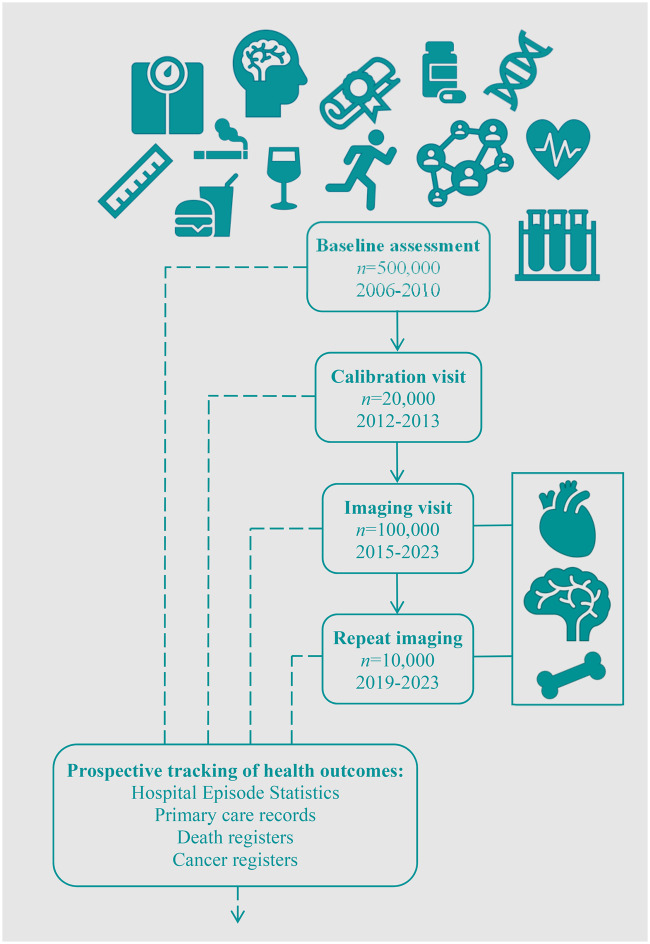
Approach to participant phenotyping in the UK Biobank.

Thus, the UKB comprises a very large sample phenotyped in great detail at multiple time-points using a variety of methods and linked to prospectively verified health outcomes (*Figure 1*), available at minimal cost to all bona fide researchers globally. The unique combination of this level of breadth, depth, and scale in a single dataset makes for a powerful research resource. In this paper, we consider how we may best utilize the cardiovascular magnetic resonance (CMR) data in conjunction with all the other information in the UKB to advance cardiovascular research and review notable achievements to date.

## The UK Biobank CMR protocol

The UKB imaging study is conducted across four UK sites (Reading, Stockport, Newcastle, Bristol) using uniform equipment, staff training, and acquisition protocols. The purpose-designed CMR protocol consists of a 20-min scan performed using a 1.5 T scanner (MAGNETOM Aera, Syngo Platform VD13A, Siemens Healthcare, Erlangen, Germany). The practical and ethical considerations posed by the large scale and observational nature of the UKB preclude the use of contrast or stress agents. The rationale, challenges, and details of the CMR protocol are described in dedicated publications.^6^^,^^7^ The protocol includes bright blood anatomic assessment (sagittal, coronal, and axial), left and right ventricular cine images (long and short axes), myocardial tagging (three short-axis slices), native T1 mapping, aortic flow, and imaging of the thoracic aorta (*Table 2*).

**Table 2:** Summary of UK Biobank cardiac magnetic resonance imaging protocol

	Sequence	Imaging planes	Related CMR indices	Clinical utility
Anatomic assessment	Bright blood, bSSFP	Sagittal, coronal, and transverse slices covering the chest and abdomen	Modified anatomic measures, e.g. aortic dimensions, lung diameters	Markers of aortic/pulmonary disease
Cardiac function	bSSFP cine	HLA, VLA, LVOT (sagittal, coronal), short-axis stack covering the right and left ventricles	RV/LV: volumes, ejection fraction, stroke volume; LV mass	Conventional markers of cardiac remodelling and function with established prognostic significance.
			Atrial size and function	Predictors of AF in the general population
			LV strain (tissue tracking)	Early marker of myocardial dysfunction
Tagging	Strain CMR (GRE)	Three short-axis slices (base, mid, and apex)	LV strain (tissue tagging)	Early marker of myocardial dysfunction
Thoracic aorta	bSSFP cine	Transverse cut at the level of the pulmonary trunk and right pulmonary artery	Aortic distensibility at the ascending and descending aorta	Markers of cardiovascular risk, in particular ischaemic disease
Aortic flow	Phase contrast flow (GRE), VENC set at 2 m/s with upward adjustment as needed	Cut plane placed at or just above the sinotubular junction at end-diastole in LVOT views (sagittal and coronal)	Aortic flow	Aortic valve anatomy and assessment of aortic stenosis
Native T1 mapping	ShMOLLI (WIP780B)	Mid-ventricular short axis	Native T1 values	Indicator of myocardial fibrosis/infarction- markers of cardiovascular disease and risk

AF, atrial fibrillation; bSSFP, balanced steady state-free precession; GRE, gradient echo; HLA, horizontal long axis; LV, left ventricle; LVOT, left ventricular outflow tract; RV, right ventricle; ShMOLLI, Shortened Modified Look-Locker Inversion recovery; VENC, velocity encoding; VLA, vertical long axis.

Conventional right and left ventricular (RV and LV) indices such as chamber volumes, ejection fraction, and LV mass may be derived from the short-axis cine stack. LV end-diastolic volume is an important indicator of adverse cardiac remodelling.^8^ Ejection fraction^9^ and LV mass^10^ are established prognostic markers. Tagging sequences allow measurement of strain, which reflects myocardial contractile function at a more granular level compared with conventional indices, such as ejection fraction.^11^ As such, alterations in myocardial strain may be appreciated at earlier or subclinical disease stages.^12^^,^^13^ Feature tracking techniques using long- and short-axis cine images are an alternative method of deriving measures of myocardial strain. They use block-matching algorithms to estimate myocardial motion by marking regions of interest along the myocardial boundaries. Feature tracking does not directly label tissue in the same way as tagging, however, post-processing is considerably faster, and estimates are adequately reliable for appreciation of associations.^14^ The long-axis cine images may also be used to obtain measures of atrial size and function, such as left atrial ejection fraction, which are reliable predictors of atrial fibrillation in the general population.^15^ This is important, as atrial fibrillation is the most common cardiac arrhythmia, particularly in older populations, with significant clinical consequences, such as the need for anticoagulation and increased risk of stroke.^16^ Native T1 mapping allows for myocardial tissue characterization without the need for contrast administration, specifically, identification of areas of fibrosis and/or infarction.^17^ Myocardial fibrosis has been linked to a number of cardiac diseases and is a marker of adverse cardiovascular outcomes such as ventricular arrhythmias and death.^18^ Infarction reflects underlying ischaemic cardiomyopathy and is also linked to increased cardiovascular risk.^19^ Aortic flow sequences permit assessment of aortic valve anatomy and function, in particular valvular stenosis. Aortic stenosis is the most common valvular pathology in older individuals, with adverse prognostic consequences and potential for alteration of its natural history with timely intervention.^20^ Aortic distensibility, a measure of vascular compliance, may be derived from transverse cine images of the thoracic aorta through consideration of the relative cross-sectional area change of the aorta (aortic strain) per unit pressure.^21^ Aortic distensibility reflects aortic bioelastic function with lower distensibility indicating a less compliant aorta and poorer vascular health.^22^ There is an inverse association between aortic distensibility and cardiovascular risk, specifically, ischaemic heart disease and stroke.^23^ Thus, aortic distensibility provides a continuous measure of ischaemic cardiovascular risk across the population.

In summary, the UKB CMR protocol provides a comprehensive assessment of cardiovascular health, providing measures of cardiac structure, function, and tissue characterization alongside multiple prognostic indices, biomarkers of subclinical disease, and indicators of important conditions such as atrial fibrillation and aortic stenosis. The CMR imaging phenotypes allow objective assessment and quantification of exposure effects on cardiovascular health and permit finer delineation of disease trajectories with potential for disease-specific assertions.

### Manual analysis of the first 5000 CMR scans

Manual segmentation of all four cardiac chambers has been completed for the first 5000 UKB CMR scans. Analysis was across two core laboratories (London, Oxford) according to a pre-defined protocol in line with international guidance.^24^ The analysis protocol is available in a separate publication.^25^ Readers across both sites received dedicated training and standardized quality control procedures were implemented. In this way, a 5000 subject manual analysis ground truth database was created. This dataset has been utilized to derive age- and sex-specific CMR normal reference ranges for the LV, RV, and atria in the largest reported cohort of validated healthy adults.^25^ The UKB CMR dataset has also resulted in a number of significant achievements providing novel insights into classical and non-classical cardiovascular risk factors, and enabling development and evaluation of novel CMR biomarkers and automated image analysis pipelines (Supplementary data online, *Table S1*).^26^

### Novel insights into classical cardiovascular risk factors

A number of researchers have used the UKB CMR dataset to provide new insights into classical cardiovascular risk factors. For instance, Petersen *et al.*^27^ define and quantify alterations in cardiac structure and function associated with known modifiable cardiovascular risk factors in individuals without pre-existing cardiovascular disease, reporting greatest effects with systolic blood pressure and body mass index. Building on these observations, Jensen *et al.*^28^ present novel insights into diabetic cardiomyopathy, demonstrating subclinical remodelling of all four cardiac chambers in diabetics without known cardiovascular disease. In a study assessing the causality of previously established associations between increased systolic blood pressure and adverse LV remodelling, Hendriks *et al.*^29^ use the genetic data in UKB to demonstrate a novel line of evidence supporting a causal relationship between elevated systolic blood pressure and higher LV mass. Linkage with the genetic data has also enabled discovery of 14 genetic loci corresponding to prognostically important LV phenotypes including end-diastolic and end-systolic volumes, mass, and ejection fraction, enhancing understanding of the genetic architecture of cardiac phenotypes and providing insights into potential novel therapeutic targets.^30^

### Investigating non-classical cardiovascular risk factors

The scale of UKB and detailed characterization of participants has enabled assessment of the effects of non-classical cardiovascular risk factors on CMR phenotypes, providing insights into novel determinants of cardiovascular disease. In a study of 1406 individuals without cardio-respiratory disease, Thomson *et al.*^31^ report association of poorer respiratory function by spirometry with adverse ventricular remodelling. Somewhat linked to these observations, Aung *et al.*^32^ report association of adverse cardiac phenotypes with past exposure to poorer air quality in 3920 individuals without clinical cardiovascular disease. Khanji *et al.*^33^ present the first study of cardiac phenotypes associated with recreational cannabis use, demonstrating larger LV volumes and impaired circumferential strain in regular cannabis users compared with never/rare users. Van Hout *et al.*^34^ consider the abdominal magnetic resonance images in UKB alongside the CMR data to investigate the relationship of body fat distribution with cardiac structure and function, demonstrating the importance of visceral obesity (vs. subcutaneous adiposity) and its association with smaller LV end-diastolic volumes and lower systolic cardiac function. In a study incorporating biochemistry, imaging, and clinical outcome data, Raisi-Estabragh *et al.*^35^ demonstrate association of poorer bone health with worse arterial health and adverse ischaemic cardiovascular outcomes and explore potential mediating mechanisms of these relationships. The UKB data has also been used to explore the association of cardiac health to other non-classical cardiovascular risk factors such as menopausal hormone therapy, spontaneous pregnancy loss, and resting heart rate.^36–38^

### Development of novel imaging biomarkers

**Figure 2:**
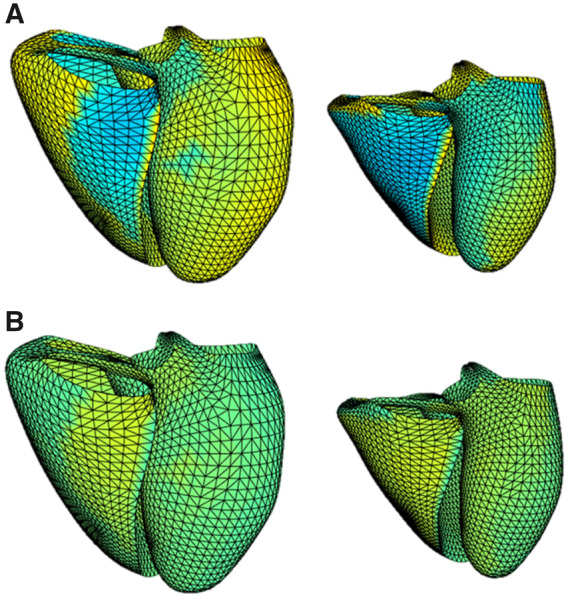
Cardiac atlas models demonstrating morphometric differences in UK Biobank participants with and without hypertension. (*A*) Hypertension and (*B*) no hypertension; models in end-diastole (left) and end-systole (right); the colours denote displacements from the mean in mm. Blue—inwards 3 mm and red—outwards 3 mm. Adapted from Ref.^42^

Several researchers have used the UKB CMR platform to investigate novel imaging biomarkers. Cardiac morphometric atlases are derived from existing CMR data and provide statistical shape models of the heart with highly detailed morphometric information.^39^ LV cardiac atlas morphometrics have been associated with a number of important cardiovascular risk factors.^40^ In the first study to compare cardiac atlases derived using different methodologies, Gilbert *et al.*^41^ use the UKB dataset to demonstrate robust associations between cardiac atlas shape measures and cardiovascular risk factors irrespective of methodology. Further, they demonstrate superior performance of cardiac atlas morphometric scores for detection of differences in LV shape associated with cardiovascular risk factors compared with conventional CMR shape indices. Building on this work, Mauger *et al.*^42^ used the UKB dataset to quantify reference RV morphometry and demonstrate complex relationships between biventricular shape and cardiovascular risk factors (*Figure 2*).

**Figure 3:**
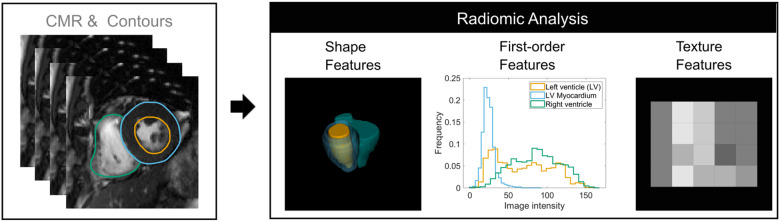
Summary of typical cardiovascular magnetic resonance radiomics feature extraction workflow. Radiomics features may be extracted from a defined region of interest. In this example, the left (orange) and right (green) ventricular endocardial and left ventricular epicardial (blue) contours are drawn in end-systole on the short-axis stack cine images. Thus, defining three regions of interest: left ventricular blood pool, right ventricular blood pool, and left ventricular myocardium. Radiomics shape features are extracted from a 3D image mask constructed from these contours. Histogram-based first-order features and more complex texture features are derived from analysis of the distribution and pattern of voxel signal intensities in the defined regions of interest. Figure courtesy of Dr Polyxeni Gkontra and Prof. Karim Lekadir, University of Barcelona.

CMR radiomics is another novel image analysis technique whereby voxel-level information is used to derive multiple quantifiers of shape and texture (*Figure 3*).^43^ There is no requirement for dedicated acquisitions or post-processing and radiomics analysis may be retrospectively applied to existing CMR images. Machine learning techniques are often used to incorporate the many extracted radiomics features (usually 100 s) as covariates into clinical prediction models. CMR radiomics models have demonstrated incremental diagnostic and predictive value in comparison to conventional methods for a number of important cardiovascular conditions.^43^ Cetin *et al.*^44^ have used data from the UKB to demonstrate the superior performance of CMR radiomics models, compared with conventional CMR indices, in discriminating individuals with hypertension from healthy comparators.

### Artificial intelligence technologies for automated image analysis

The large volume of data in the UKB image bank necessitates the development of automated image analysis pipelines that are scalable, require minimal manual interaction, and have standardized quality control measures. The 5000 reference cohort and their corresponding contours have enabled development and evaluation of machine learning methods for cardiac chamber segmentation with some promising results.^45^ In particular, Attar *et al.*^46^ present a fully automatic pipeline performing end-to-end analytics from cine images to anatomic and functional quantification (LV and RV) on 20 000 UKB CMR scans validated against the ground truth cohort of manual segmentations. A fully automated image analysis tool for measurement of aortic distensibility has also been developed and validated on a large subset of UKB studies (*n *=* *5100); the analysis pipeline can detect and locate aortic areas and has in-built quality control mechanisms.^47^

In addition to these purpose-built pipelines, fully automated LV quantification is performed as part of UKB image acquisitions using the Siemens syngo InlineVF software (Siemens Healthcare, Erlangen, Germany, version D13A). The InlineVF analysis algorithm determines the LV endocardial contours on the short-axis slices, defines the LV base (mitral valve) and apex on long-axis slices, and outputs standard LV indices (volumes, ejection fraction, stroke volume). Whilst raw results from this analysis are provided by the UKB, the InlineVF software is intended for use in clinical settings with expert assessment of contour quality. Therefore, it is advisable to apply quality control measures to the fully automated outputs of UKB. After formal evaluation of the InlineVF outputs, we recommend that these be used with implementation of visual assessment for quality control and linear bias correction.^48^

### Potential for future work

To best utilize the UKB, we must consider the resource in its entirety and appreciate the complementary value of its different components. The scale and extensive participant phenotyping in UKB permits consideration of a large number of exposures and their potential interactions with many disease conditions. These research opportunities will increase as incident disease outcomes accrue and the imaging study is completed. The breadth, depth, and scale of phenotypic information in UKB also yield unique opportunities to investigate relationships of risk factors acting across organ systems. There is increasing interest in exploration of cross-system interactions with notable work exploring the heart–brain^49^ and heart–gut^50^ axes. Already, researchers have demonstrated links between cognition and structural brain MRI features and cardiac health in the UKB.^51^ As disease outcomes accrue within the UKB cohort, there will be greater opportunity to explore these important cross-system interactions.

The large standardized UKB imaging dataset provides an ideal platform for development and evaluation of automated image analysis pipelines. Artificial intelligence technologies for high volume image phenotype extraction could translate readily to clinical settings, improving time and resource efficiency. Substantial progress has been made with automated extraction of conventional ventricular indices and aortic distensibility in the UKB. Similar work is underway to develop scalable automated processes for analysis of tagging, native T1 mapping, tissue tracking, and aortic flow sequences. These areas have not yet been published on and are ripe for exploration. The dataset is also the ideal setting for development of novel CMR biomarkers. In addition to providing a platform for technical development, linkage to participant characteristics and outcomes uniquely enables assessment of clinical utility within the same sample.

## Conclusions

The UKB presents the opportunity to examine prospectively, in a single, robustly powered, and characterized cohort, a wide range of exposure-outcome relationships and the potential interactions between them. As incident health outcomes accrue, and the imaging study is completed, UKB will offer huge opportunities to undertake highly powered studies to comprehensively investigate the determinants of cardiovascular disease. It is now up to the imagination and expertise of researchers to translate this unique resource into real benefits for our patients and thus reduce the burden of cardiovascular disease worldwide.

## Supplementary data

Supplementary data are available at *European Heart Journal - Cardiovascular Imaging* online.

## Funding

Z.R.-E. was supported by a British Heart Foundation Clinical Research Training Fellowship (FS/17/81/33318). N.C.H. acknowledges support from the UK Medical Research Council, NIHR Southampton Biomedical Research Centre, University of Southampton, and University Hospital Southampton. S.E.P. acknowledges support from the National Institute for Health Research (NIHR Biomedical Research Centre at Barts), the ‘SmartHeart’ EPSRC programme grant (www.nihr.ac.uk; EP/P001009/1), and the European Union’s Horizon 2020 research and innovation programme (825903). S.N. was supported by the Oxford NIHR Biomedical Research Centre and the Oxford British Heart Foundation Centre of Research Excellence.

**Conflict of interest:** S.E.P. acts as a paid consultant to Circle Cardiovascular Imaging Inc., Calgary, Canada, and Servier.

## Supplementary Material

jeaa297_Supplementary_Data

## References

[1] UK Biobank: Protocol for a Large-Scale Prospective Epidemiological Resource, 2007 https://www.ukbiobank.ac.uk/wp-content/uploads/2011/11/UK-Biobank-Protocol.pdf (29 September 2020, date last accessed).

[2] UK Biobank Imaging Study. https://imaging.ukbiobank.ac.uk/ (29 September 2020, date last accessed).

[3] Schnier C , Sudlow Biobank CU. Algorithmically-Defined Health Outcomes (Chief Scientist), with Input from Members of the UK Biobank Follow-up and Outcomes Adjudication Group, 2017 https://biobank.ctsu.ox.ac.uk/crystal/crystal/docs/alg_outcome_main.pdf (29 September 2020, date last accessed).

[4] Manolio TA , Collins R. Enhancing the feasibility of large cohort studies. JAMA Amer Med Assoc 2010;304:2290–1. 10.1001/jama.2010.1686 PMC3075846 21098774

[5] Fry A , Littlejohns TJ, Sudlow C, Doherty N, Adamska L, Sprosen T et al Comparison of sociodemographic and health-related characteristics of UK Biobank participants with those of the general population. Am J Epidemiol 2017;186:1026–34. 28641372 10.1093/aje/kwx246 PMC5860371

[6] Petersen SE , Matthews PM, Bamberg F, Bluemke DA, Francis JM, Friedrich MG et al Imaging in population science: cardiovascular magnetic resonance in 100,000 participants of UK Biobank – rationale, challenges and approaches. J Cardiovasc Magn Reson 2013;15:46. 23714095 10.1186/1532-429X-15-46 PMC3668194

[7] Petersen SE , Matthews PM, Francis JM, Robson MD, Zemrak F, Boubertakh R et al UK Biobank’s cardiovascular magnetic resonance protocol. J Cardiovasc Magn Reson 2015;18:8. 10.1186/s12968-016-0227-4 PMC4736703 26830817

[8] Gjesdal O , Bluemke DA, Lima JA. Cardiac remodeling at the population level—risk factors, screening, and outcomes. Nat Rev Cardiol 2011;8:673–85. 22027657 10.1038/nrcardio.2011.154

[9] Curtis JP , Sokol SI, Wang Y, Rathore SS, Ko DT, Jadbabaie F et al The association of left ventricular ejection fraction, mortality, and cause of death in stable outpatients with heart failure. J Am Coll Cardiol 2003;42:736–42. 12932612 10.1016/s0735-1097(03)00789-7

[10] Bluemke DA , Kronmal RA, Lima JAC, Liu K, Olson J, Burke GL et al The relationship of left ventricular mass and geometry to incident cardiovascular events. J Am Coll Cardiol 2008;52:2148–55. 19095132 10.1016/j.jacc.2008.09.014 PMC2706368

[11] Amzulescu MS , De Craene M, Langet H, Pasquet A, Vancraeynest D, Pouleur AC et al Myocardial strain imaging: review of general principles, validation, and sources of discrepancies. Eur Hear J Cardiovasc Imaging 2019;20:605–19. 10.1093/ehjci/jez041 PMC6529912 30903139

[12] Buss SJ , Breuninger K, Lehrke S, Voss A, Galuschky C, Lossnitzer D et al Assessment of myocardial deformation with cardiac magnetic resonance strain imaging improves risk stratification in patients with dilated cardiomyopathy. Eur Heart J Cardiovasc Imaging 2015;16:307–15. 25246506 10.1093/ehjci/jeu181

[13] Delgado V , Tops LF, Van Bommel RJ, Van der Kley F, Marsan NA, Klautz RJ et al Strain analysis in patients with severe aortic stenosis and preserved left ventricular ejection fraction undergoing surgical valve replacement. Eur Heart J 2009;30:3037–47. 19726436 10.1093/eurheartj/ehp351

[14] Claus P , Omar AMS, Pedrizzetti G, Sengupta PP, Nagel E. Tissue tracking technology for assessing cardiac mechanics: principles, normal values, and clinical applications. JACC Cardiovasc Imaging 2015;8:1444–60. 26699113 10.1016/j.jcmg.2015.11.001

[15] Olsen FJ , Møgelvang R, Jensen GB, Jensen JS, Biering-Sørensen T. Relationship between left atrial functional measures and incident atrial fibrillation in the general population: the Copenhagen City Heart Study. JACC Cardiovasc Imaging 2019;12:981–9. 29454773 10.1016/j.jcmg.2017.12.016

[16] John Camm A. Atrial fibrillation and risk. Clin Cardiol 2012;35:S1–2. 10.1002/clc.21961 PMC6652333 22246945

[17] Haaf P , Garg P, Messroghli DR, Broadbent DA, Greenwood JP, Plein S. Cardiac T1 mapping and extracellular volume (ECV) in clinical practice: a comprehensive review. J Cardiovasc Magn Reson 2017;18:89. 10.1186/s12968-016-0308-4 PMC5129251 27899132

[18] Taylor AJ , Salerno M, Dharmakumar R, Jerosch-Herold M. T1 mapping basic techniques and clinical applications. JACC Cardiovasc Imaging 2016;9:67–81. 26762877 10.1016/j.jcmg.2015.11.005

[19] Baxa J , Ferda J, Hromádka M. T1 mapping of the ischemic myocardium: review of potential clinical use. Eur J Radiol 2016;85:1922–8. 27105590 10.1016/j.ejrad.2016.04.010

[20] Joseph J , Naqvi SY, Giri J, Goldberg S. Aortic stenosis: pathophysiology, diagnosis, and therapy. Am J Med 2017;130:253–63. 27810479 10.1016/j.amjmed.2016.10.005

[21] Resnick LM , Militianu D, Cunnings AJ, Pipe JG, Evelhoch JL, Soulen RL. Direct magnetic resonance determination of aortic distensibility in essential hypertension. Hypertension 1997;30:654–9. 9322999 10.1161/01.hyp.30.3.654

[22] Laurent S , Cockcroft J, Van Bortel L, Boutouyrie P, Giannattasio C, Hayoz D, on behalf of the European Network for Non-invasive Investigation of Large Arteries et al Expert consensus document on arterial stiffness: methodological issues and clinical applications. Eur Heart J 2006;27:2588–605. 17000623 10.1093/eurheartj/ehl254

[23] Said Eppinga AM , Lipsic RN, Verweij E, Van der Harst N. P. Relationship of arterial stiffness index and pulse pressure with cardiovascular disease and mortality. J Am Heart Assoc 2018;7:e007621. 29358193 10.1161/JAHA.117.007621 PMC5850166

[24] Schulz-Menger J , Bluemke DA, Bremerich J, Flamm SD, Fogel MA, Friedrich MG et al Standardized image interpretation and post processing in cardiovascular magnetic resonance: Society for Cardiovascular Magnetic Resonance (SCMR) Board of Trustees Task Force on Standardized Post Processing. J Cardiovasc Magn Reson 2013;15:35. 23634753 10.1186/1532-429X-15-35 PMC3695769

[25] Petersen SE , Aung N, Sanghvi MM, Zemrak F, Fung K, Paiva JM et al Reference ranges for cardiac structure and function using cardiovascular magnetic resonance (CMR) in Caucasians from the UK Biobank population cohort. J Cardiovasc Magn Reson 2017;19:18. 28178995 10.1186/s12968-017-0327-9 PMC5304550

[26] Raisi-Estabragh Z , Petersen SE. Cardiovascular research highlights from the UK Biobank: opportunities and challenges. Cardiovasc Res 2020;116:e12–5. 31778147 10.1093/cvr/cvz294

[27] Petersen SE , Sanghvi MM, Aung N, Cooper JA, Paiva JM, Zemrak F et al The impact of cardiovascular risk factors on cardiac structure and function: insights from the UK Biobank imaging enhancement study. PLoS One 2017;12:e0185114. 28973022 10.1371/journal.pone.0185114 PMC5626035

[28] Jensen MT , Fung K, Aung N, Sanghvi MM, Chadalavada S, Paiva JM et al Changes in cardiac morphology and function in individuals with diabetes mellitus. Circ Cardiovasc Imaging 2019;12:e009476. 31522551 10.1161/CIRCIMAGING.119.009476 PMC7099857

[29] Hendriks T , Said MA, Janssen LMAA, Van der Ende MY, Van Veldhuisen DJ, Verweij N et al Effect of systolic blood pressure on left ventricular structure and function. Hypertension 2019;74:826–32. 31476911 10.1161/HYPERTENSIONAHA.119.12679

[30] Aung N , Vargas JD, Yang C, Cabrera CP, Warren HR, Fung K et al Genome-wide analysis of left ventricular image-derived phenotypes identifies fourteen loci associated with cardiac morphogenesis and heart failure development. Circulation 2019;140:1318–30. 31554410 10.1161/CIRCULATIONAHA.119.041161 PMC6791514

[31] Thomson RJ , Aung N, Sanghvi MM, Paiva JM, Lee AM, Zemrak F et al Variation in lung function and alterations in cardiac structure and function—analysis of the UK Biobank cardiovascular magnetic resonance imaging substudy. PLoS One 2018;13:e0194434. 29558496 10.1371/journal.pone.0194434 PMC5860758

[32] Aung N , Sanghvi MM, Zemrak F, Lee AM, Cooper JA, Paiva JM et al Association between ambient air pollution and cardiac morpho-functional phenotypes: insights from the UK Biobank Population Imaging Study. Circulation 2018;138:2175–86. 30524134 10.1161/CIRCULATIONAHA.118.034856 PMC6250297

[33] Khanji MY , Jensen MT, Kenawy AA, Raisi-Estabragh Z, Paiva JM, Aung N et al Association between recreational cannabis use and cardiac structure and function. JACC Cardiovasc Imaging 2020;13:886–8. 31864983 10.1016/j.jcmg.2019.10.012

[34] Van Hout MJP , Dekkers IA, Westenberg JJM, Schalij MJ, Scholte AJHA, Lamb HJ. The impact of visceral and general obesity on vascular and left ventricular function and geometry: a cross-sectional magnetic resonance imaging study of the UK Biobank. Eur Hear J Cardiovasc Imaging 2020;21:273–81. 10.1093/ehjci/jez279 PMC7031704 31722392

[35] Raisi‐Estabragh Z , Biasiolli L, Cooper J, Aung N, Fung K, Paiva JM et al Poor bone quality is associated with greater arterial stiffness: insights from the UK Biobank. J Bone Miner Res 2020. doi:10.1002/jbmr.4164. [online ahead of print] 10.1002/jbmr.4164 PMC7613252 32964541

[36] Sanghvi MM , Aung N, Cooper JA, Paiva JM, Lee AM, Zemrak F et al The impact of menopausal hormone therapy (MHT) on cardiac structure and function: insights from the UK Biobank imaging enhancement study. PLoS One 2018;13:e0194015. 29518141 10.1371/journal.pone.0194015 PMC5843282

[37] Elmahi E , Sanghvi MM, Jones A, Aye CYL, Lewandowski AJ, Aung N et al Does self-reported pregnancy loss identify women at risk of an adverse cardiovascular phenotype in later life? Insights from UK Biobank. Kirchmair R, ed. PLoS One 2019;14:e0223125. 31644534 10.1371/journal.pone.0223125 PMC6808447

[38] Raisi-Estabragh Z , Cooper J, Judge R, Khanji MY, Munroe PB, Cooper C et al Age, sex and disease-specific associations between resting heart rate and cardiovascular mortality in the UK Biobank. PLoS One 2020;15:e0233898. 32470091 10.1371/journal.pone.0233898 PMC7259773

[39] Young AA , Frangi AF. Computational cardiac atlases: from patient to population and back. Exp Physiol 2009;94:578–96. 19098087 10.1113/expphysiol.2008.044081

[40] Medrano-Gracia P , Cowan BR, Ambale-Venkatesh B, Bluemke DA, Eng J, Finn JP et al Left ventricular shape variation in asymptomatic populations: the multi-ethnic study of atherosclerosis. J Cardiovasc Magn Reson 2014;16:56. 25160814 10.1186/s12968-014-0056-2 PMC4145340

[41] Gilbert K , Bai W, Mauger C, Medrano-Gracia P, Suinesiaputra A, Lee AM et al Independent left ventricular morphometric atlases show consistent relationships with cardiovascular risk factors: a UK Biobank Study. Sci Rep 2019;9:1130. 30718635 10.1038/s41598-018-37916-6 PMC6362245

[42] Mauger C , Gilbert K, Lee AM, Sanghvi MM, Aung N, Fung K et al Right ventricular shape and function: cardiovascular magnetic resonance reference morphology and biventricular risk factor morphometrics in UK Biobank. J Cardiovasc Magn Reson 2019;21:41. 31315625 10.1186/s12968-019-0551-6 PMC6637624

[43] Raisi-Estabragh Z , Izquierdo C, Campello VM, Martin-Isla C, Jaggi A, Harvey NC et al Cardiac magnetic resonance radiomics: basic principles and clinical perspectives. Eur Hear J Cardiovasc Imaging 2020;1–8. 10.1093/ehjci/jeaa028 PMC7082724 32142107

[44] Cetin I , Petersen SE, Napel S, Camara O, Ballester MAG, Lekadir K. A radiomics approach to analyse cardiac alterations in hypertension. Int Symp Biomed Imaging 2019;640–3.

[45] Bai W , Sinclair M, Tarroni G, Oktay O, Rajchl M, Vaillant G et al Automated cardiovascular magnetic resonance image analysis with fully convolutional networks. J Cardiovasc Magn Reson 2018;20:65. 30217194 10.1186/s12968-018-0471-x PMC6138894

[46] Attar R , Pereañez M, Gooya A, Albà X, Zhang L, de Vila MH et al Quantitative CMR population imaging on 20,000 subjects of the UK Biobank imaging study: LV/RV quantification pipeline and its evaluation. Med Image Anal 2019;56:26–42. 31154149 10.1016/j.media.2019.05.006

[47] Biasiolli L , Hann E, Lukaschuk E, Carapella V, Paiva JM, Aung N et al Automated localization and quality control of the aorta in cine CMR can significantly accelerate processing of the UK Biobank population data. PLoS One 2019;14:e0212272. 30763349 10.1371/journal.pone.0212272 PMC6375606

[48] Suinesiaputra A , Sanghvi MM, Aung N, Paiva JM, Zemrak F, Fung K et al Fully-automated left ventricular mass and volume MRI analysis in the UK Biobank population cohort: evaluation of initial results. Int J Cardiovasc Imaging 2018;34:281–91. 28836039 10.1007/s10554-017-1225-9 PMC5809564

[49] Lyall DM , Cullen B, Allerhand M, Smith DJ, Mackay D, Evans J et al Cognitive test scores in UK Biobank: data reduction in 480,416 participants and longitudinal stability in 20,346 participants. PLoS One 2016;11:e0154222. 27110937 10.1371/journal.pone.0154222 PMC4844168

[50] Tang WHW , Kitai T, Hazen SL. Gut microbiota in cardiovascular health and disease. Circ Res 2017;120:1183–96. 28360349 10.1161/CIRCRESAHA.117.309715 PMC5390330

[51] Cox SR , Lyall DM, Ritchie SJ, Bastin ME, Harris MA, Buchanan CR et al Associations between vascular risk factors and brain MRI indices in UK Biobank. Eur Heart J 2019;40:2290–300. 30854560 10.1093/eurheartj/ehz100 PMC6642726

