# Supplementary material for: Cardiovascular magnetic resonance imaging in the UK Biobank: a major international health research resource
Source: Eur Heart J Cardiovasc Imaging. 2020 Nov 9;22(3):251–8. doi: 10.1093/ehjci/jeaa297 (PMC7899275; doi:10.1093/ehjci/jeaa297)
Supplement: jeaa297_Supplementary_Data [file jeaa297_supplementary_data.docx]

**Supplementary Table 1. Summary of selected studies using UK Biobank CMR data**

| Author, year of publication | Research question/aim | Methods | Summary of findings |
| --- | --- | --- | --- |
| Raisi-Estabragh et al. 2020 | What is the association of speed of sound from quantitative heel ultrasound with measures of arterial stiffness (AoD, ASI) and with ischaemic cardiovascular outcomes and what are the mediating factors? | Mutivariable linear regression models of the association between speed of sound and ASI/AoD. Cox/competing risk regression models to test association of speed of sound with incident myocardial infarction and ischaemic heart disease death. Subanalysis by sex and menopause status. Multiple mediation analysis to examine mediating effect of a range of biochemical variables. | Findings support a positive association between bone and vascular health with consistent patterns of association in men and women. The underlying mechanisms are complex and appear to vary by sex. |
| Hout et al. 2020 | What is the association of body fat distribution with cardiovascular structure and function? | Multivariable regression models were used to test the association of subcutaneous adiposity, visceral adiposity and body fat percentage with CMR cardiovascular phenotypes in 4,590 UKB participants. | Visceral obesity was associated with a smaller LV EDV and subclinical lower LV systolic function in men, suggesting that visceral obesity might play a more important role compared to general obesity in LV remodelling. |
| Biasiolli et al. 2019 | To develop and validate a fully automated method to detect and localise the ascending and descending aorta for AoD measure with a quality control mechanism. | The automated AA and PDA detection-localization algorithm followed these steps: 1) foreground segmentation; 2) detection of candidate ROIs by Circular Hough Transform; 3) spatial, histogram and shape feature extraction for candidate ROIs; 4) AA and PDA detection using Random Forest (RF); 5) quality control based on RF detection probability. The algorithm was tested on 3,900 UKB CMR scans. | The proposed method for automated AA and PDA localization was extremely accurate and the automatically derived detection probabilities provided a robust mechanism to detect low quality scans for further human review. |
| Attar et al. 2019 | To develop and evaluate a fully automated CMR image analysis pipeline. | The authors present and evaluate a fully automatic scalable CMR image analysis pipeline with inbuilt quality control using 20,000 cases from the UKB for LV/RV quantification. The pipeline is validated on 4,620 manually annotated UKB cases. | The presented pipeline performs end-to-end image analytics from multi-view cine CMR to LV/RV quantification without need for manual user interactions, with quality control of image input and outputted segmentations. |
| Jensen et al. 2019 | What are the early alterations in cardiac structure and function associated with DM? | Multivariable regression models were built to ascertain the association of DM status with CMR phenotypes in a subpopulation without pre-existing cardiovascular disease and LVEF $\geq$ 50% (n=3984) | In a low-risk general population without known cardiovascular disease and with preserved LV ejection fraction, DM was associated with early changes in all 4 cardiac chambers. |
| Hendriks et al. 2019 | What are the effects of lifelong exposure to high SBP on LV structure and function? | A genetic risk score to estimate genetically predicted SBP (gSBP) was constructed based on 107 previously established genetic variants. Manual CMR image analysis was performed for 300 individuals at the extremes of gSBP. Multivariable linear regression analyses of imaging biomarkers were performed using gSBP as continuous independent variable. | This study provides a novel line of evidence for a causal relationship between SBP and increased LV mass and with increased LV global radial strain. |
| Aung et al. 2019 | What is the genetic basis of LV image-derived phenotypes? | Genome wide association study of LVEDV, LVESV, LVEF, and LVM, using 16,923 CMR cases from the UK Biobank and genotyping data at baseline. | 14 novel genetic loci were identified for LV CMR phenotypes. |
| Khanji et al. 2019 | What is the association of cannabis use with cardiovascular structure and function on CMR? | Multivariate regression models were used to test effect of regular, never/rare, or previous cannabis use on CMR cardiovascular indices in a sample of 3,407 UKB participants. | Regular cannabis use was associated with larger LVEDV, LVESV, and impaired global circumferential strain compared with rare/no cannabis use. |
| Elmahi et al. 2019 | What is the association between history of pregnancy loss and imaging measures of cardiovascular function? | Multivariable linear regression models were used to test association between self-reported pregnancy loss and CMR measures of cardiac structure and function and carotid ultrasound measures of arterial health in 2660 women from UKB. | In this analysis, women who self-report pregnancy loss did not have significant differences in cardiac structure, cardiac function, or carotid structure in later life. |
| Gilbert et al. 2019 | What are the associations of cardiac atlas morphometric measures with cardiovascular risk factors and do these vary by type of atlas? | Two independent LV atlases were constructed from 4,547 UKB CMR scans. The strength of associations between atlas principal components and cardiovascular risk factors (smoking, DM , high blood pressure, high cholesterol and angina) were quantified with logistic regression models. Comparison was made between different atlases. | Morphometric variations associated with each risk factor could be quantified and visualized and were similar between atlases. UK Biobank LV shape atlases are robust to construction method and show stronger relationships with cardiovascular risk factors than mass and volume. |
| Mauger et al. 2019 | What are the associations between cardiovascular disease risk factors and the biventricular cardiac atlas morphometrics? | A biventricular shape atlas was automatically constructed using contours and landmarks from 4,329 UKB CMR studies. A reference sub-cohort was identified consisting of 630 participants with no cardiovascular risk factors. Morphometric scores were computed using linear regression to quantify shape variations associated with high cholesterol, high blood pressure, obesity, smoking, DM, previous myocardial infarction and angina. | Morphometric relationships between biventricular shape and cardiovascular risk factors in a large cohort show complex interactions between RV and LV morphology. These can be quantified by z-scores, which can be used to study the morphological correlates of disease. |
| Bai et al. 2018 | To develop automated methods for CMR cardiac chamber segmentation? | Aa fully convolutional network was trained and evaluated on a 4,875 CMR studies from the UK Biobank to develop a fully an automated analysis method for segmentation of LV, RV, LA, and RA. | The presented automated method achieves a performance on par with human experts in analysing CMR images and deriving clinically relevant measures. |
| Sanghvi et al. 2018 | What is the effect of menopausal hormonal therapy on CMR cardiovascular phenotypes? | Multivariable linear regression was performed to examine the relationship between CMR cardiac parameters and menopausal hormonal therapy use $\geq$ 3 years in 1,604 postmenopausal women from UKB. | Menopausal hormonal therapy use was not associated with adverse, subclinical changes in cardiac structure and function |
| Aung et al. 2018 | What is the effect of exposure to ﻿ambient air pollution on CMR cardiovascular phenotypes? | Multivariable linear models were built to test association of previous exposure to ambient air pollution on CMR indices of cardiac structure and function in 3,920 UKB participants without pre-existing cardiovascular disease. | Higher past exposure to particulate matter with an aerodynamic diameter <2.5 µm and nitrogen dioxide was associated with cardiac ventricular dilatation, a marker of adverse remodelling. |
| Thomson et al. 2018 | What is the relationship between lung function and CMR cardiovascular phenotypes in individuals without respiratory disease? | Multivariable linear models were built to test association of spirometry measures of lung function (obtained at baseline UKB visit) with CMR indices of cardiac structure and function in individuals without respiratory disease (n=1,406) | This study shows that reduced FEV1 and FVC are associated with smaller ventricular vol- umes and reduced ventricular mass. The changes seen per standard deviation change in FEV1 and FVC are comparable to one decade of ageing. |
| Petersen et al. 2017 | What is the impact of classical cardiovascular risk factors on cardiac CMR phenotypes? | Multivariable regression models were built to ascertain the association of risk factors (Age, sex, ethnicity, SBP, DBP, smoking status, exercise, BMI, high cholesterol, DM, alcohol intake) on LV, RV, LA and RA CMR parameters in 4,651 UKB participants. | Modifiable risk factors are associated with subclinical alterations in structure and function in all four cardiac chambers. BMI and SBP were the most important factors affecting CMR parameters known to be linked to adverse outcomes. |

Supplementary Table 1 caption: AA: ascending aorta; AoD: aortic distensibility; ASI: arterial stiffness index; BMI: body mass index; CMR: cardiovascular magnetic resonance; DM: diabetes mellitus; EDV: end-diastolic volume; FEV1: forced expiratory volume 1; FVC: forced vital capacity; LA: left atrium; LV: left ventricle; LVEF: left ventricular ejection fraction; LVM: left ventricular mass; PDA: proximal descending aorta; RA: right atrium; ROI: region of interest; RV: right ventricle; SBP: systolic blood pressure; UKB: UK Biobank
